# Circadian Levels of Serum Melatonin and Cortisol in relation to Changes in Mood, Sleep, and Neurocognitive Performance, Spanning a Year of Residence in Antarctica

**DOI:** 10.1155/2013/254090

**Published:** 2012-12-13

**Authors:** Madhumita Premkumar, Tarulata Sable, Dinesh Dhanwal, Richa Dewan

**Affiliations:** ^1^National Centre for Antarctic and Ocean Research, Ministry of Earth Sciences, Headland Sada, Goa 403 804, India; ^2^Department of Medicine, B.L. Taneja Block, Maulana Azad Medical College, Bahadur Shah, Zafar Marg, New Delhi 110002, India

## Abstract

*Background*. Altered circadian cortisol and melatonin rhythms in healthy subjects exposed to an extreme polar photoperiod results in changes in mood and sleep, which can influence cognitive performance. *Materials and Methods*. We assessed the circadian rhythm of 20 subjects who wintered over at Maitri (70°S, 11°E), India's permanent Antarctic station, from November 2010 to December 2011. Serum cortisol and melatonin levels were measured by radioimmunoassay at 8 am, 3 pm, 8 pm, and 2 am in a single day, once each during the polar summer and winter photoperiods. Conventional psychological tests, Depression, Anxiety, and Stress Scale (DASS-42), Epworth Sleepiness Scale (ESS), and a computerized neurocognitive test battery were used to measure mood, sleep, and cognitive performance. *Results*. The mean scores for DASS42 were higher during midwinter suggesting the presence of “overwintering.” Mean diurnal cortisol levels during summer and winter were comparable, but the levels of melatonin were markedly higher during winter. Higher 8 am melatonin levels were associated with better sleep quality, lower depression scores, and better performance in tasks like attention, visual memory, and arithmetic. *Conclusion*. Timing of artificial light exposure and usage of melatonin supplements in improving sleep and cognitive performance in expedition teams are of future research interest.

## 1. Introduction

Residence in Antarctica exposes humans working in the research bases to extremes of weather and photoperiod, in addition to geographic and social isolation. The annual average solar radiation decreases with increasing latitude, and this difference is marked during winter than in midsummer in the circumpolar regions. This alters temporal homeostasis and affects sleep wake cycles, mood, cognition and work performance of base personnel. The psychological disturbances in Antarctica, referred to as the “over winter syndrome,” may also relate to change in the circadian rhythms of the hormones cortisol and melatonin [1–3]. Photic stimuli are the primary synchronizer of the endogenous circadian pacemaker in the suprachiasmatic nucleus of the anterior hypothalamus. Therefore temporal modulation of the ambient light settings over a 24 hour period in Antarctica can promote better adjustment to the seasonal photoperiod. The endocrine control over the circadian pacemaker is reflected by the changes in serum levels of cortisol and the pineal indoleamine melatonin. Melatonin, noradrenaline, and acetylcholine decrease with light activation, whereas cortisol, serotonin, GABA, and dopamine levels increase. In normal subjects, we expect a peak melatonin level at approximately 3 am, whereas cortisol peaks at approximately 9 am [4]. In healthy individuals, the transition from low light to bright light in the morning results in a surge in cortisol levels and a decline in melatonin levels [5]. Since the photoperiod is the principal factor regulating seasonal adaptation, melatonin responses may mediate changes in endocrine, cognitive, psychological, and immune function [6]. 

There is mounting evidence to suggest that long-term disruption of circadian rhythm correlates with disease states such as ischemic stroke [7], heart disease [8], metabolic dysfunction, and glucose intolerance [9]. Additional studies have also found that chronic shift work is significantly associated with an increased risk of colorectal, endometrial, and prostate cancers [10]. Disintegration of sleep wake cycles and poor sleep health is also associated with a range of neuronal diseases such as Alzheimer's or Huntington's disease [11, 12]. Psychological research in Antarctica provides insight into how individuals (and groups) function in an extreme and isolated milieu. However, while interpreting and comparing published data, the racial, psychosocial, educational and cultural differences of expedition teams needs to be considered. 

Sleep disturbances and psychological changes have been reported in overwintering teams, but only one study on 9 individuals has tested the effect of the changed diurnal rhythm in the Antarctic on human cognition and performance [13]. Previous studies have reported that circadian cortisol levels have an important role in cognition in an age independent manner. Studies demonstrated that frequent transmeridian flyers with a disrupted diurnal cycle had higher evening cortisol levels, which was associated with cognitive deficits and right temporal lobe atrophy [14]. Objective assessment of cognitive performance is important for comparison and reproducibility. Computerized neurocognitive tests (CNT) allow accurate measurements of reaction times and test-retest reliability. However, they may be less reliable than conventional tests when traits like verbal skills and personality are being tested [15]. Hence a combined approach is suitable in situations where comprehensive evaluation of personality and serial testing of cognitive skills is needed.

The objective of this study was to (i) evaluate the seasonal variation in serum cortisol and melatonin diurnal rhythms during the polar summer and winter photoperiods, (ii) study mood and depression, and (iii) assess the change in objective neurocognitive performance of the base personnel over a period of one year in Antarctica.

## 2. Materials and Methods

### 2.1. Subjects and Study Design

Twenty male members of the 30th Indian Scientific Expedition to Antarctica (ISEA), certified to be in good mental and physical health and free of intrinsic sleep disorders following medical and psychiatric examination and acclimatization training, were recruited for this prospective study with written informed consent. The study was approved by an Institutional Ethics Board of the National Centre for Antarctic and Ocean Research (NCAOR), India's nodal agency for Antarctic research and was conducted in accordance with the standards of the Declaration of Helsinki. The study was started in November 2010 soon after arrival of team members in Antarctica and the group was followed up prospectively till the conclusion of the expedition in December 2011.

### 2.2. Study Protocol

The Indian Antarctic research base Maitri is located at 70°45′57′′S, 11°44′09′′E. The over-wintering team consisted of 6 scientists and 14 technical support staff who are required to keep the base functional and the scientific experiments running. All the personnel are required to maintain the same sleep rhythm (wake at 0700 hours UTC) and work schedule except for one person who is on night watch, which is a periodic duty once every ten days by rotation. One meteorologist also maintained a night shift duty for the greater part of the year.

The winter period at 70°S starts in mid April and lasts till October. Serum levels of melatonin and cortisol were measured in the subjects at 8 am, 3 pm, 8 pm, and 2 am in a single day using an indwelling antecubital catheter during 2 different photoperiods; once during summer and the other during the polar winter. The samples reflecting the summer photoperiod were collected in January 2011, a few weeks after arrival in Antarctica, and processed to obtain serum which was stored at minus 25° Celsius until assay was done. Repeat serum samples were collected in June 2011 during the polar winter. The serum was frozen immediately and stored at minus 25°C. The samples were transported back to India on dry ice using monitored cold chain transport at −25°C to −70°C. 

Melatonin was measured by radioimmunoassay (RIA), using I^125^-melatonin (Labor Diagnostika Nord GmbH & Co. KG, Nordhorn, Germany). The reference range, as stated by the manufacturer, was 30–150 pg/mL. The sensitivity of the method was 2.3 pg/mL and the intra-assay variability less than 9.8% and inter-assay variability 8%. The antimelatonin antibody used had a cross reactivity of >0.1% with melatonin related metabolites or precursors. Serum levels of cortisol were assayed by GammaCoat RIA (DiaSorin, Stillwater, MN) with a sensitivity of 0.2 *μ*g/dL and intra-assay coefficient of variation (CV) of 6.5% and inter-assay variability of 9%. The reference range was 7–25 *μ*g/dL for morning values and 2–9 *μ*g/dL for evening values.

### 2.3. Sleep Quality Evaluation

All subjects were required to maintain a sleep log over a 2 month period in summer and again in winter. The sleep log documented the sleep/wake times, caffeine and alcohol intake, night time awakenings, use of sleep aids such as sleeping pills, music, reading, total duration of sleep, and a subjective evaluation of restorative sleep. Epworth Sleepiness Scale (ESS) [16] and Morningness Eveningness Questionnaire (MEQ) [17] were administered on arrival, during summer and during winter. The ESS is a validated scale for measuring daytime sleepiness and the MEQ scores reveal individual adjustment to morning and evening photoperiods and the synchronization of the internal clock to work schedules.

### 2.4. Neurocognitive Evaluation

A combination of conventional psychological tests and a computerized neurocognitive test battery (CNT) were used to serially measure relatively mild degrees of neurocognitive impairment in the base personnel. CNTs were performed using Psychology Experiment Building Language (PEBL) Version 0.11. The tests included visual and verbal memory, attention, visual motor speed, arithmetic and reaction times, a modification of the Halstead Reitan Battery, to test domains likely to be relevant to wintering and working in a challenging environment like Antarctica (see Table 1).

Neurocognition Tests: the battery of tests employed in this study included both conventional and computerized tests [15].

#### 2.4.1. Attention

(1) *Card Sort Test (CST)*. The card sort test measures shifting attention, that is, the ability of the subject to focus on one set of instructions and then to shift to another set in response to the change in test response. The test involves 4 packs of cards which have different geometric shapes (circle, star, triangle, and cross), different number of shapes (1, 2, 3, and 4) and different colours (red, yellow, green, and blue). Then a card with a pattern having a specific colour and number of shapes is presented and the subject is instructed to match the card with one of the 4 packs by a rule which needs to be deduced from the test response. The rule is changed randomly (matching of colour, number, or shape) throughout the test and the subject is scored for the number of correct matches made in a fixed time. This is a modification of the Wisconsin Card Sort Test and measures attention and cognitive flexibility [18]. 

(2) *Digit Span Forward*. Random digits (0 through 9) are presented to the subject on the screen and simultaneously by computer generated voice in increasing sequence as long as correct responses are obtained. If the response is incorrect, the subject is tested with a shorter sequence which is then increased by one digit successively until the longest sequence of digits is correctly repeated. The test is continued for 20 trials and the mean number and longest sequence of correct responses is calculated. 

(3) *Continuous Performance Test (CPT)*. This test measures sustained attention or task related vigilance. The subject is instructed to press the space bar when any alphabet is flashed on the screen except the alphabet “X.” The reaction time for each target stimulus is measured. The test is performed with 300 trials of 16 blocks. The entire test takes about 14 minutes. The number of “X” foil stimuli is 25 and target alphabet stimuli are 300, respectively. The test gives the accuracy rates for the targets and foil non targets and also the mean reaction times for correct and erroneous responses. This is a sensitive test for central nervous system dysfunction [15]. 

#### 2.4.2. Executive Function

*Stroop Test*. The subject is presented with four colors/color words (red, green, blue, yellow) and is instructed to select either the colour or the word. At first 5 practice trials are allowed. Then 20 random trials for congruent and incongruent colour words are presented. In total 5 words are presented in a congruent color and word (e.g., word RED in red font) which generates a congruent reaction time and 15 test words are presented in an incongruent fashion (e.g., word RED in green font), which generates a second reaction time [15]. 

#### 2.4.3. Visual Motor Ability

*Finger Tapping Test*. The subject is asked to press the key “X” on the computer keyboard as many times as possible in 90 seconds. This is recorded separately for the right and left index finger. This simple test provides reliable and reproducible data about fine motor control, motor speed, and visual motor function [19]. 

#### 2.4.4. Visual Memory Test

The visual memory test involves the presentation of a sequence of everyday object images with their names (apple, pear, bus, bird, etc.) on the screen. Each image is flashed for 2 seconds in increasing number and the subject has to memorize the order of presentation. Then a screen of nine figures arrayed in a grid is presented and the subject is instructed to click on each image in the order of presentation. The test is repeated with 3 trial blocks of up to 9 image sequences. It generates the average number of images remembered correctly in any sequence. This is based on the Rey Visual Design learning test [20]. 

#### 2.4.5. Arithmetic

The subject is presented with simple addition, subtraction, multiplication, and division equations (up to 2 integers) at first slowly and then with increasing frequency for a period of 3 minutes. The score is based on the accuracy and number of equations solved per minute.

#### 2.4.6. Speed of Processing

(1) *Simple Reaction Time*. The subjects were instructed to click the left mouse button each time a triangle was flashed on the screen for ten trials. This generates the longest, shortest, and mean reaction times. A similar test for auditory reaction time was used by presenting a tone stimulus by headphones. The test generates the longest, shortest, and mean reaction times. 

(2) *Choice Reaction Time*. The subjects were presented an image of a geometric design (circle, square, triangle, oval) and another image at random immediately after. If the new image was identical to the one immediately before it, they were asked to click the right shift button and if not, the left shift button. The reaction times and accuracy was recorded over 1 minute for a sequence of random images [21]. 

(3) *Psychological Tests*. The instruments for assessing physical and mental health included demographic information on age, occupation, education, and subjective health complaints, and standardized scales such as depression, anxiety, and stress scale (DASS-42) [22] and Mini Mental Status Examination (MMSE) [23]. DASS-42 measures the behavioral manifestations of depression, anxiety, and stress in a self report interview to construct a score (see Table 2).

Period differences in the circadian rhythm were tested by measuring serum cortisol and melatonin levels during summer and winter. The sampling periods were correlated with the ambient solar radiation over the station during the polar summer and winter photoperiods. This was measured by means of a sunlight photometer during the subjects' sojourn in Antarctica, and data was obtained with permission of the Indian Meteorological Department (IMD).

### 2.5. Statistical Analyses

Conventional methods, mean and standard deviation (SD), Student's *t*-test, Fisher's *T* test, and Pearson's correlation coefficient were used to analyze the diurnal variation in cortisol and melatonin levels with the level of significance <0.05. All data were reported as means ± SD. The software SPSS for Windows ver.13 (SPSS Inc; Chicago, IL, USA; http://www.spss.com/) was used for statistical analysis.

## 3. Results

The mean age of the 20 male subjects was 39 ± 10.24 years. All the support staff had a high school education and an additional technical qualification. Eighteen (90%) of the 20 subjects were right handed. The entire team was subjected to psychological testing prior to selection for the expedition and none of them suffered from depression or sleep disorders.

Table 3 shows the basic clinical characteristics of the subjects. There was a trend for an increased weight, BMI, and systolic blood pressure in older individuals. Four (20%) were hypertensive, requiring antihypertensive drugs in addition to lifestyle and diet modification. Two of these subjects were newly diagnosed with the disease in Antarctica. 

### 3.1. Psychological Changes

The maximum score of the DASS-42 is 42 in each of the depression, anxiety, and stress subscales. Therefore lower scores are better.  Table 4 showing the DASS42 subscale scores is revealing. The mean scores for depression and anxiety during midwinter were significantly higher than summer scores (*P* < 0.005) suggesting the presence of “over wintering.” Daytime sleepiness was excessive (score 16) in just one subject as per the ESS, but 4 subjects had borderline scores (mean 11.4 ± 1.1) during summer and 2 of them continued to report daytime sleepiness during midwinter.

### 3.2. Sleep Quality and Subjective Polar Acclimatization

The work schedule during summer for the technical and maintenance staff involved reporting for duty at 0900 hrs and mild to moderate physical work for a period of 3-4 hours. Post lunch (1230–1400 hrs) there was another work period from 1500 to 1800 hrs. With the onset of the polar night, the evening shift was stopped due to adverse weather conditions and poor visibility. However during blizzards, special rotation duties for snow clearance and station maintenance resulted in a peak in physical activity. These periods of 2–4 days were also associated with increases in perceived stress. The scientists maintained the same work schedule in summer and winter, but participated in external work and blizzard duties. Additionally, all team members participated in “galley” duties by rotation involving night watch every 8–10 days.

The sleep logs showed a marked difference between summer and winter periods in 5 (25%) subjects, all scientists. They reported a decreased latency, increased duration of sleep, fewer early morning awakenings, and restorative sleep as compared to summer. The sleep patterns of the technical support team varied only in the total duration of sleep during winter (mean 8.42 ± 1.18 hours) as compared with summer (mean 8.64 ± 1.21 hours). In contrast, the total sleep duration in scientists was 7.33 ± 0.98 hours in summer and 7.66 ± 1.03 hours in winter (see Table 5). The total duration of sleep was increased due to an increased frequency of afternoon napping which was related more to no work shift in the post lunch period rather than due to increased fatigue. Sleep aids used by the subjects included listening to music, reading, and watching television. Pharmacological therapy, including use of benzodiazepines and zolpidem, was needed in 7 (35%) subjects at one or more times for sleep induction.

### 3.3. Cortisol and Melatonin Diurnal Rhythm

**Figure 1:**
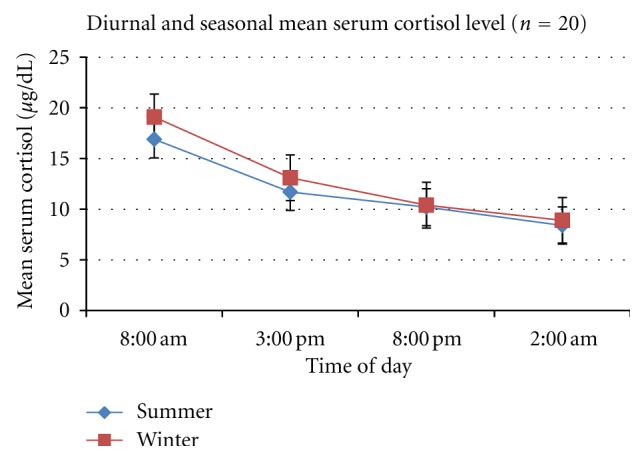
Diurnal variation of mean serum cortisol levels of 20 subjects with standard error (SEM).

**Figure 2:**
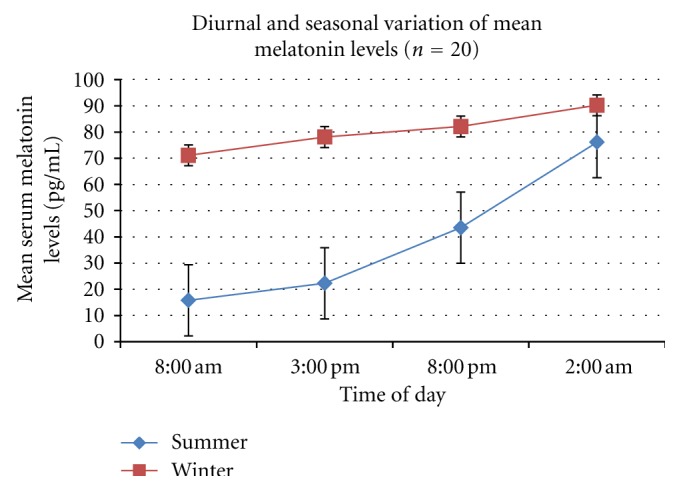
Diurnal variation of mean serum melatonin levels of 20 subjects with standard error (SEM).

 The results of the diurnal and seasonal variation of cortisol and melatonin provide interesting biochemical correlates for our clinical data. As expected, the mean cortisol levels were highest at 8 am, with a progressive decline during the day, the nadir being reached at 2 am. The mean levels in winter were higher at 8 am and 3 pm but the difference was statistically equivalent (see Figure 1). During midwinter, we noted a marked increase in the mean serum melatonin levels. Again, the maximum levels were noted at 2 am which is physiological. But melatonin levels were persistently high throughout the day.  Figure 2 demonstrates the marked contrast in melatonin diurnal rhythm during the two seasonal photoperiods. While the mean levels of these hormones showed a physiological and predictable diurnal rhythm, we noted marked individual variation. This was more so for scientific staff involved in time bound data collection. 

The scores on MEQ provided clues to the degree of subjective acclimatization in relation to polar work and photoperiod. Soon after arrival and during midsummer, 5 (25%) subjects preferred starting work late between 10 and 11 am and up to 4 (20%) persons responded that they were mentally and physically more active in the evening. Twelve (60%) had a “definitely morning,” chronotype, 2 (10%) corresponded to “moderately morning” and 4 (20%) were neither type. In contrast, during midwinter, 14 (70%) subjects favored a “definitely morning” chronotype as opposed to a “moderately morning” 4 (20%) or “neither morning nor evening” chronotype 2 (10%). Most reported a preference to start work earlier, between 8 and 9 am. 

We assessed whether cortisol and melatonin diurnal rhythms correlated with psychological tests and cognitive performance (see Tables 6 and 7). In most cases, no clear trend was obtained for hormone levels and cognitive function. We did find that a higher evening cortisol negatively correlated with performance on tests for attention like digit span and card sort during summer. Higher evening cortisol levels also correlated with increased DASS 42 scores. During winter, elevated 8 am melatonin levels correlated significantly with better performance on tests for attention, digit span, and arithmetic during winter. Although this may seem paradoxical, an increased 8 am melatonin level was also associated with better sleep quality, which may have improved performance status.

### 3.4. Neurocognitive Assessment

The subjects were tested by both conventional methods and by computerized tests in the morning. Those with a “non morning” chronotype were retested in the evening, but there was no significant change in the scores other than the continuous performance test (CPT) for attention. The results of the mean scores and reaction times are shown in Table 8. Overall, the mean scores and reaction times of many tests were statistically equivalent in the summer and winter photoperiods. There was a significant increase in arithmetic computational accuracy and speed in winter. Also subjects performed better on choice reaction time with lower mean RT and improved accuracy suggesting improvement in speed of processing. There was a marginal improvement in scores of the card sort test for shifting attention and digit span in winter. This was more so for individuals with higher 8 am melatonin levels (*P* = 0.034). However CNT results need to be interpreted in the appropriate clinical context. Subjects with higher DASS-42 scores, scored lower on digit span (*P* = 0.032), shifting attention (*P* = 0.045), and CPT (*P* = 0.012). Mean scores for simple reaction time, digit span, and visual memory were better in individuals aged <30 years. Higher ESS scores did not correlate with poor performance on CPT, and no “sleep attacks” were noted during testing.

## 4. Discussion

Circadian rhythms are intrinsic to all living systems. Adaptation to the polar environment involves synchronization to temporally relevant stimuli such as light, temperature, work, meal schedules and thus the internal clock gets aligned with the external solar time. Rhythmic modulation of behavior and physiologic processes such as sleep, cardiovascular, cognitive performance results from the circadian control over diverse pathways [25]. 

### 4.1. Circadian Rhythm in Antarctica

Few studies have been done on circadian rhythm in Antarctica and no study has ever been done on the impact of prolonged Antarctic residence on circadian rhythm in Indian expedition team members [1–3, 26, 27]. Sleep schedules, activity, plasma melatonin, and core body temperature were documented in 9 Japanese subjects at Dome station (77°S). They concluded that the sleep rhythm was largely governed by the work schedule while the circadian rhythm in plasma and core body temperature is influenced by photoperiod [1]. Adaptation of the melatonin rhythm in British Antarctic personnel at Halley station (75°S) has also been reported in night shift workers over short periods [2]. Another study also evaluated the usage of light intensity regulation at the stations to influence sleep behavior and mood disturbance issues. They concluded that further investigations of phase shifting techniques such as timed bright light and administration of melatonin are indicated to improve performance at work [3, 13].

A recent study of salivary cortisol awakening response in 55 participants at 2 British Antarctic stations Rothera (67°34′S, 68°8′W) and Halley (75°35′S, 26°30′W) showed no difference in the diurnal cortisol response in the polar winter. They also reported depression in only 7% individuals [28]. 

We measured the serum levels of cortisol and melatonin during summer about a month after arrival in Antarctica. The period of transition from one expedition team to the next involves adjustment to new routines, constant daylight and cold dry windy weather. This high activity period results in an elevated stress hormone profile. Therefore the effect of adaptation stress must be kept in mind while interpreting results. But we found that the mean 8 am cortisol levels were higher in winter when compared with summer. An increased 8 am summer cortisol level correlated with a reduced reaction time (*P* = 0.018). Subjects showing a higher 8 am melatonin level during winter also performed better in the neurocognition tests for immediate memory (*P* = 0.034), and arithmetic (*P* = 0.041), suggesting better adaptation. This may seem counterintuitive at first glance, as melatonin is a hypnotic hormone. The result may be best interpreted in the context of restorative sleep improving cognitive performance in winter. An elevated evening cortisol did not correlate with deficits in visual recognition memory, in contrast with previous studies. High evening cortisol levels may indicate chronic stress and poor sleep quality, which in turn impairs performance [28, 29]. In our study, no clear association could be obtained for individual cortisol or melatonin values and the corresponding neurocognitive test results of the subjects. This is largely due to the smaller sample size, which is restricted by the number of team members in an over wintering expedition. So results need to be interpreted carefully, as multiple factors may affect an individual's day to day performance.

### 4.2. Sleep Quality

There was an improvement in sleep quality, duration, and decreased latency in winter. The maximum sleep disturbances occurred in the first one month after arrival, in the phase of adjustment to a 24 hour light photoperiod. The scientific staff had fewer total sleep hours than the logistic staff and also reported increased sleep latency. The sleep wake cycles of scientists were irregular, changing with night watch, galley duties, and timed data collection. Subjects also reported better sleep quality, fewer night awakenings, and decreased latency during winter which correlated with higher melatonin levels. Studies from other stations reported that poor sleep was associated with depressive symptoms. In our study, patients with higher DASS-42 scores reported night interruptions and early awakening. Winter was also associated with an increased volume and frequency of alcohol intake, but no changes in sleep parameters were associated with drinking. Sleep aids are useful and pharmacological therapy is often needed in the early adjustment phase to avoid sleep disruption.

### 4.3. Circadian Rhythm and Neurocognitive Performance in Antarctica

The subtle changes in behavior, sleep, memory, and cognition during long duration expeditions may have long term health consequences on aging, cardiovascular, and cerebrovascular disease. In this study, we performed tests for domains like memory, attention, reaction time, and speed of processing which can be affected by extrinsic factors like drugs, disease states, and time of day testing. Also, motivation, interest, mood, and vigilance affect individual scores. Though the cognitive performance for the whole team averaged the same during summer and winter, we noted other outcomes. Subjects with depression had lower scores for memory and shifting attention in midwinter. This may reflect subtle changes in the level of alertness as a function of diurnal rhythm. Despite advances of technology and upgraded facilities at scientific bases, it remains a challenge to winter over in the Antarctic. Subjects have to perform energy consuming tasks including but not limited to snow clearance, prolonged driving, and maintenance of vehicles, heating systems, electrical generators, and so forth. These activities are prone to human error. In addition interpersonal relationships and closed group psychology and the sense of physical isolation affect performance [30–32]. 

## 5. Conclusions

 Circadian rhythms are modulated by central pacemakers like melatonin and cortisol which synchronize to stimuli such as photoperiod and activity, and thus indirectly the internal clock becomes predictive of solar time. Our study found that the diurnal rhythm for cortisol was comparable during the polar summer and winter. In contrast, circadian secretion of melatonin was affected during the polar winter and higher levels persisted throughout the day, peaking physiologically at 2 am in both seasons. This was associated with better subjective sleep quality during winter. Again, higher levels of 8 am melatonin correlated significantly with better neurocognitive performances in tests for attention and immediate memory. We also report the effect of over wintering in our subjects reflected as higher depression, anxiety, and stress scale scores during the polar winter. This impaired cognitive performance in few subjects. In light of the fact that the team size of wintering teams in the Antarctic is kept to a minimum, pooling of data will be useful in making recommendations for future expeditions. Timing of artificial light exposure and usage of melatonin supplements in improving sleep and cognitive performance in expedition teams are of future research interest.

## Figures and Tables

**Table 1:** Neurocognitive domains and tests used in the study protocol.

Serial number	Domain	Neurocognitive Test	References

1	Attention/concentration	Shifting attention test	
		Digit span forward	Tulsky et al. [18]
		Continuous performance test	
2	Executive function	Stroop test	Tornatore et al. [24]
3	Visual motor ability	Index finger tapping test (FTT)	Gill et al. [19]
4	Visual memory	Modified Rey Visual Design Learning test	Shapiro and Harrison [20]
5	Arithmetic	Speed and accuracy of simple addition,	
		subtraction, multiplication and division equations.	
6	Speed of processing	Simple and choice reaction time	Lemay et al. [21]

**Table 2:** Psychological tests used in the study protocol and interpretation of scores.

S. no	Scale	Grading scale	Number of questions	Interpretation of score	

1	DASS 42	0 = does not apply	42		Normal
		1 = applied to some degree		Depression	0–9
		2 = applied to a considerable degree		Anxiety	0–7
		3 = applied very much		Stress	0–14

		0 = would never doze		0–10 Normal range	
2	ESS	1 = Slight chance	8	10–12 Borderline	
		2 = Moderate chance		12–24 Abnormal	
		3 = High chance of dozing	

3	MEQ	Provides a “chronotype” of the individual	19	(1) Definitely Morning type = 64–56	
				(2) Moderately morning type = 54–46	
				(3) Neither type = 44–32	
				(4) Moderately evening type = 30–22	
				(5) Definitely evening type = 20–12	

4	MMSE		30	Normal 27–30.	

DASS-42: depression, anxiety, and stress scale; ESS: Epworth sleepiness scale; MEQ: Morningness Eveningness Questionnaire; MMSE: Mini mental status examination.

**Table 3:** Clinical profile of subjects on arrival, during summer, and during winter.

Age (yrs)	Subjects (*n*)	Body Weight (kg)	BMI (kg/m^2^)	Mean systolic BP (mmHg)			Mean diastolic BP (mmHg)		
Mean ± standard deviation									
				Arrival	Summer	Winter	Arrival	Summer	Winter

20–29	5 (25%)	65.4 ± 21.57	23.18 ± 6.82	123.0 ± 20.85	117.46 ± 12.83	117.46 ± 17.04	77.6 ± 8.29	77.2 ± 5.22	73.73 ± 9.91
30–39	4 (20%)	69 ± 1.33	24.63 ± 1.25	128.0 ± 5.42	121.83 ± 5.21	119.83 ± 9.20	82.5 ± 5.42	79.83 ± 6.63	76.5 ± 6.03
40–49	8 (40%)	76.13 ± 11.43	27.74 ± 4.30	93.25 ± 6.7	130.66 ± 12.58	126.91 ± 12.18	76.13 ± 11.43	83 ± 5.74	78.75 ± 7.86
>50	3 (15%)	83 ± 15.39	28.69 ± 2.19	96.0 ± 12.16	138 ± 16.21	131.33 ± 16.18	83 ± 15.39	88.22 ± 7.90	82 ± 9.59

**Table 4:** Mean scores, standard deviation, and percentage of personnel reporting depression, anxiety, stress (DASS-42), and daytime sleepiness (ESS) and the corresponding mini mental status (MMSE) score during summer, winter, and before departure.

		Summer		Winter				Departure	
S. no	Scale	Mean ± SD	*N* (%) with	Mean ± SD	*N* (%) with	*P *	Effect	Mean ± SD	*N* (%) with
			abnormal score		abnormal score	value^*£*^	Size (*d*)^†^		abnormal score

1	DASS 42								
	*Depression *	5.0 ± 1.07	0 (0%)	7.7 ± 3.2	4 (20%)	*0.002 *	−1.131	6.45 ± 2.35	2 (10%)
	* Anxiety *	6.4 ± 0.99	11 (55%)	7.6 ± 1.56	11 (55%)	*0.005 *	−0.918	6.85 ± 1.64	5 (40%)
	* Stress *	11.5 ± 4.24	4 (20%)	14.4 ± 4.86	10 (20%)	*0.040 *	−0.635	12.4 ± 4.13	6 (30%)
2	ESS	7.95 ± 3.01	4 (20%)	9.4 ± 2.47	7 (35%)	*0.04 *	−0.526	8.45 ± 2.13	5 (40%)
3	MMSE	28.4 ± 1.84	—	28.25 ± 1.88	—	*0.72 *	0.080	28.15 ± 1.66	—

^*£*^Paired sample *T* test for significant difference of means between summer and winter values. ^†^Cohen's *d* for effect size.

DASS 42: depression anxiety and stress scale; SD: standard deviation; ESS: Epworth sleepiness scale; MMSE: Mini mental status examination.

**Table 5:** Subjective assessment of sleep parameters during the summer and winter photoperiods in Antarctica.

	Summer	Winter	*P* value

Latency (min)	19.25 ± 9.35	16 ± 10.07	0.003
Duration (hrs)	8.1 ± 1.28	8.35 ± 1.03	0.204
Restorative sleep	11 (55%)	17 (85%)	
Early awakenings	7 (35%)	6 (30%)	
% reporting awakenings at night	8 (40%)	4 (20%)	
Splitting sleep schedule	6/20 (30%)	2/20 (10%)	
Subjective sleep quality; *n* (%)			
(i) Good	11 (55%)	11 (55%)	
(ii) Fair	5 (25%)	7 (35%)	
(iii) Poor	4 (20%)	2 (10%)	
Sleep aids *n* (%)	4/20 (25%)	6/20 (30%)	
Caffeine intake (average number of cups/day)	2.6	2.7	0.329
Mean alcohol intake (g/week)	59.81 (*n* = 11)	88.5 (*n* = 12)	0.002

**Table 6:** Correlation of serum cortisol levels with clinical parameters, psychological tests, and neurocognitive performance during the polar summer and winter photoperiods.

S. No	Parameter/Test	Summer				Winter			
		Pearson's correlation coefficient *r *							
		8 am	3 pm	8 pm	2 am	8 am	3 pm	8 pm	2 am

1	Age	−0.192	0.079	−0.139	−0.112	−0.305	0.316	−0.182	−0.254
2	Weight	0.242	−0.326	−0.218	0.125	0.203	0.015	−0.064	0.066
3	BMI	0.434	−0.123	−0.236	0.328	0.342	0.108	0.154	0.652
4	DASS 42	0.047	−0.126	−0.012	0.264	0.308	0.579∗	0.184	0.438∗
5	ESS	−0.864∗	−0.042	0.053	0.036	−0.034	0.030	0.014	0.272
6	MEQ	−0.618	0.114	−0.346	−0.543∗	−0.034	−0.169	0.031	0.912
7	MMSE	0.101	−0.346	−0.025	−0.584∗	−0.102	0.595∗	0.354	−0.001
8	Attention								
	Card sort test (correct response)	0.120	−0.025	−0.782∗	−0.342	0.013	0.153	0.217	0.248
	Digit span	−0.019	−0.879∗	−0.145	0.312	−0.168∗	0.336	0.085	0.058
	Continuous performance test (target accuracy rate)	0.147	0.096	0.764	0.342	0.143	0.314	0.018	0.214
9	Executive function Stroop test (mean reaction time)	0.367	0.453	0.032	0.672	0.156	0.098	0.542	0.345
10	Visual motor ability FTT	0.519∗	0.378	0.462	0.176	0.135	0.619	0.503	0.913
11	Visual memory	−0.045	0.147	0.134	0.439	0.400	−0.336	0.136	0.305
12	Arithmetic equations per minute	0.183	−0.025	0.156	0.283	0.050	0.054	0.148	0.177
13	Speed of processing mean simple RT	−0.519∗	0.295	0.341	0.175	0.083	−0.349	0.053	0.065
	Mean choice RT	0.321	0.346	0.145	0.467	−0.413	0.143	0.401	0.312

BMI: body mass index; FTT: finger tapping test; RT: reaction time. ∗Significant at *P* < 0.05.

**Table 7:** Correlation of diurnal serum melatonin levels with clinical parameters, psychological test results, and neurocognitive performance during the polar summer and winter photoperiods.

S. No	Parameter/Test	Summer				Winter			
		Pearson's correlation coefficient *r *							
		8 am	3 pm	8 pm	2 am	8 am	3 pm	8 pm	2 am

1	Age	−0.244	−0.150	−0.333	0.159	−0.057	−0.127	0.072	−0.454
2	Weight	−0.128	−0.113	−0.225	0.137	0.124	−0.287	−0.118	0.170
3	BMI	0.217	0.014	−0.213	−0.214	0.343	0.129	−0.412	0.125
4	DASS 42	0.099	0.268	−0.207	0.047	−0.122	0.603∗	−0.130	0.375
5	ESS	−0.328	0.024	0.246	0.179	0.044	−0.104	0.102	0.068
6	MEQ	0.123	0.332	0.312	0.221	−0.212	−0.123	0.213	0.251
7	MMSE	−0.353	−0.012	−0.080	−0.672∗	−0.009	−0.392∗	0.114	−0.244
8	Attention								
	Card sort test (correct response)	−0.220	0.134	0.401	−0.002	0.347∗	0.219	0.014	−0.013
	Digit span	−0.280	0.107	0.127	0.901∗	0.306∗	−0.271	0.120	0.813
	Continuous performance test (target accuracy rate)	−0.219	0.061	0.312	0.208	0.143	0.247	0.134	0.287
9	Executive function Stroop test (mean reaction time)	0.309	0.149	−0.213	0.134	0.103	−0.124	0.217	0.219
10	Visual motor ability FTT	0.012	0.312	0.204	0.132	0.139	0.412	−0.132	−0.012
11	Visual memory	0.029	−0.134	0.013	0.210	0.403	0.410	0.176	0.289
12	Arithmetic (equations per minute)	−0.247	−0.143	0.124	−0.065	0.451∗	−0.002	−0.166	0.316
13	Speed of processing mean simple RT	0.360	0.312	0.132	0.466∗	−0.113	−0.314	0.304	0.289
	Mean choice RT	0.115	0.198	−0.312	0.113	−0.213	0.274	0.149	0.163

BMI: body mass index; FTT: finger tapping test; RT: reaction time. ∗Significant at *P* < 0.05.

**Table 8:** Results of neurocognitive assessment during the polar summer and winter.

	Number of participants (*n* = 20)	Summer	Winter		
S. no	Domain/Cognitive Test	Mean ± SD	Mean ± SD	*P* value^∧^	Effect size (*d*)^†^

1	Attention				
	1.1 Card sort test (CST)				
	CST correct	48.98 ± 7.94	50.22 ± 7.52	0.038	−0.160
	CST errors	11.52 ± 4.27	11.10 ± 3.96	0.340	0.101
	CST reaction time (ms)	1109.43 ± 196.66	1108.56 ± 208.83	0.959	0.004
	1.2 Digit span	5.98 ± 0.69	6.08 ± 0.73	0.258	−0.140
	1.3 Continuous performance test (CPT)				
	CPT target accuracy rate	0.97 ± 0.02	0.98 ± 0.02	0.514	−0.500
	CPT foil accuracy rate	0.76 ± 0.12	0.74 ± 0.13	0.379	0.159
	CPT correct RT (ms)	438.78 ± 37.78	433.28 ± 43.16	0.705	0.135
	CPT error RT (ms)	350.11 ± 103.38	336 ± 91	0.329	0.144
2	Executive function				
	Stroop test				
	Stroop congruent RT (ms)	97.39 ± 32.95	94.59 ± 31.63	0.404	0.086
	Stroop incongruent RT (ms)	105.87 ± 34.78	105.316 ± 33.28	0.876	0.016
3	Visual motor ability				
	FTT right (taps/10 seconds)	47.84 ± 7.12	48.14 ± 7.65	0.725	−0.040
	FTT left (taps/10 seconds)	39.19 ± 6.72	39.34 ± 7.22	0.854	−0.020
4	Visual memory	6.3 ± 2.2	6.4 ± 1.96	0.343	−0.021
5	Arithmetic				
	Mean total equations solved (accuracy %)	47.1 ± 15.24 (91.45%)	55.35 ± 14.66 (77.95%)	0.014	−0.551
	Equations per minute	13 ± 3.98	16.35 ± 3.75	0.000	−0.788
6	Speed of processing				
	6.1 Mean simple RT (s)	0.4205 ± 0.102	0.4355 ± 0.100	0.387	−0.148
	Long SRT (s)	0.5218 ± 0.195	0.5194 ± 0.167	0.969	0.013
	Short SRT (s)	0.2980 ± 0.075	0.2894 ± 0.056	0.649	0.135
	6.2 Mean choice RT (ms)	2037.95 ± 542.36	1508.2 ± 320.02	0.000	1.189
	Correct trials	19.5 ± 5.55	32.9 ± 7.35	0.170	−1.625
	Accuracy rate of CRT (%)	89.1 ± 8.85%	92.68 ± 22.73%	0.214	−0.207

RT: reaction time; SRT: simple reaction time; CRT: choice reaction time; FTT: finger tapping test; SD: standard deviation.

^*∧*^Paired sample *t* test for difference of means significant at *P* < 0.05.

^†^Effect size using Cohen's *d* test.
